# A Rare Phenomenon of Natural Precocious Mummification

**DOI:** 10.7759/cureus.45626

**Published:** 2023-09-20

**Authors:** Biliana Mileva, Ivan Tsranchev, Mihaela Georgieva, Metodi Goshev, Alexandar Alexandrov

**Affiliations:** 1 Department of Forensic Medicine and Deontology, Medical University Sofia, Sofia, BGR; 2 Department of Forensic Medicine and Deontology, Medical University of Plovdiv, Plovdiv, BGR

**Keywords:** forensic entomology, post-mortem interval, decomposition, mummification, precocious

## Abstract

Immediately after death, specific changes occur in the human body, leading to the total dissolution of the soft tissues and internal organs. In some cases, when in suitable conditions, the decomposition process could stop and be displaced by mummification. The last one is time-consuming and needs several weeks to months to set in completely. We present a case of a 34-year-old man found dead 16 days after being last seen alive in a stage of complete mummification. Natural mummification occurring in less than one month is termed precocious mummification and is rarely observed in temperate regions. With only a few cases reported globally, this case is essential for the forensic community. It will help better know the mummification processes and estimate the time since death.

## Introduction

The mummification process of human bodies (natural or artificial and complete or partial) is known from ancient times [1]. Mummification is a postmortem process that preserves the body and is associated with total drying of the soft tissues, giving the skin a so-called leathery appearance [2]. Many factors could affect the mummification process, but the most important ones are dry air, good ventilation, solar radiation, and high temperature [3,4]. Depending on the conditions, the time for complete mummification of the human body could vary widely. Still, this process usually takes several weeks to months [2,4].

## Case presentation

**Figure 1:**
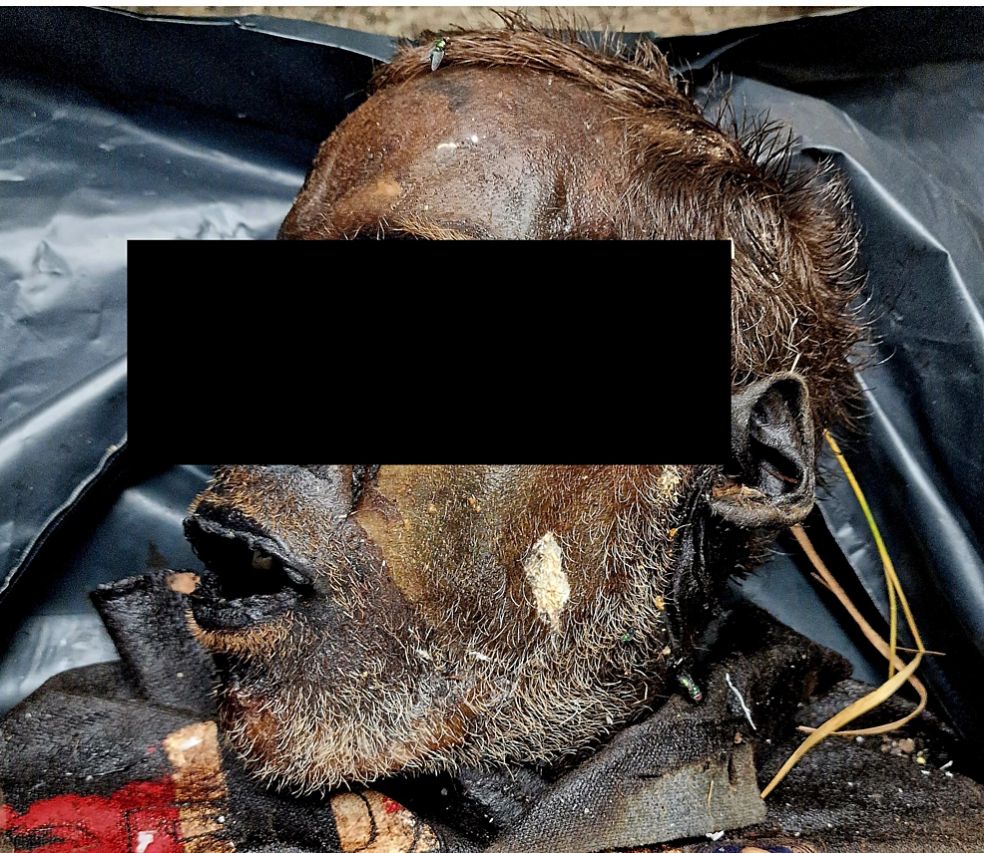
External examination of the corpse – mummified facial skin

On the 3rd of September 2023 in Sofia, Bulgaria, a human body was found in the lawns next to a railway line. Next to the body was his bag, packed with personal stuff and a bottle of alcoholic beverage. The police found out that the body was of a 34-year-old man, who was last seen alive on the 18th of August, 2023. The corpse was wearing a t-shirt, shorts, and socks, and it was confirmed that those were the clothes with which he was last seen alive. There was information that the deceased was chronically abusing alcohol. The body was sent for an autopsy at the Clinic of Forensic Medicine and Deontology, Sofia. During the external examination, no traumatic injuries were found. The skin surface showed coloration ranging from light to dark brown, and it was hard and leathery (Figure 1).

**Figure 2:**
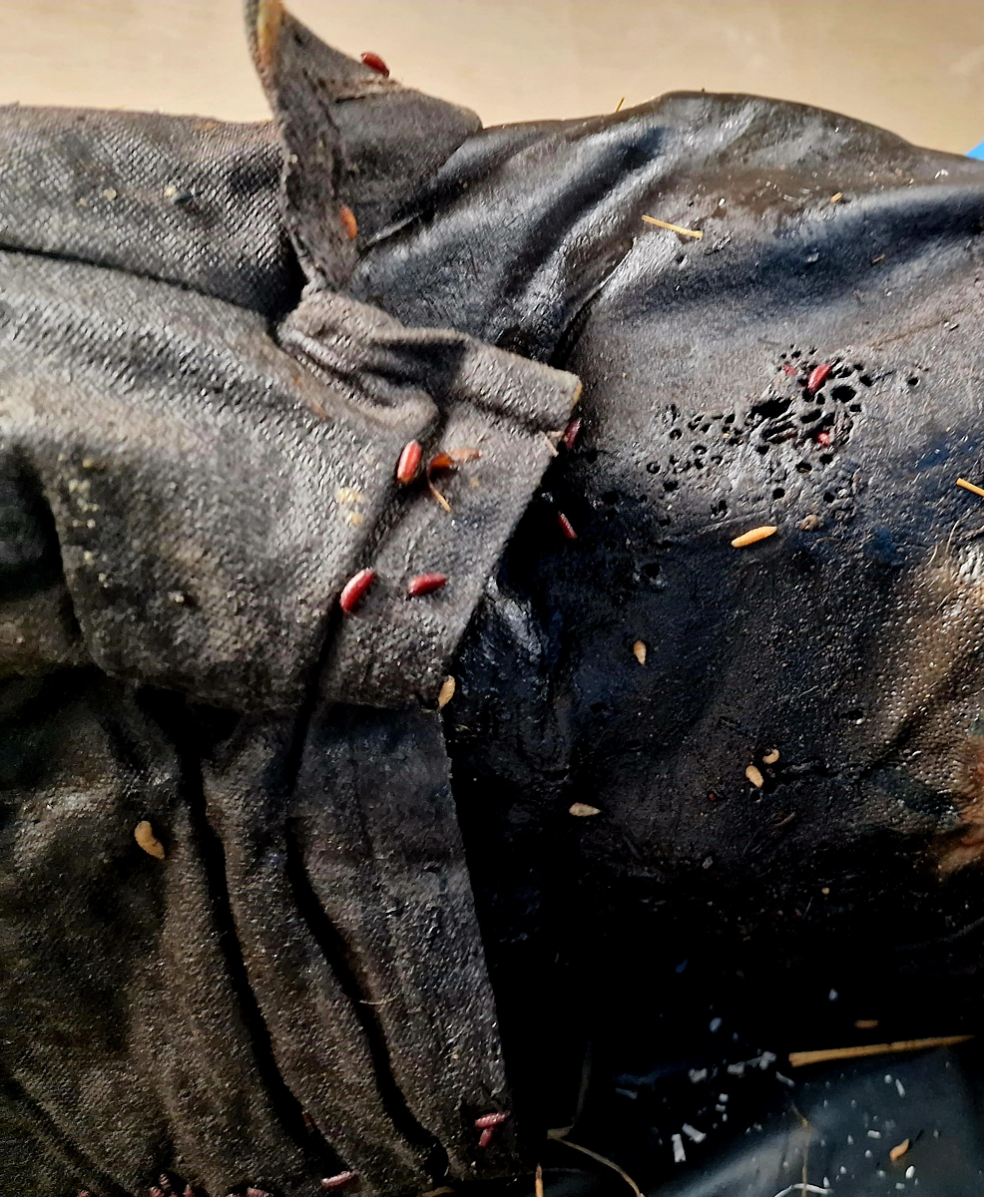
Mummified skin of the pelvic area with roundish lesions from maggots – with larvae and pupas

On different sides of the body, multiple small roundish lesions were present, consistent with postmortem activity from maggots. The body was covered with a small amount of maggots varying from 1-2 mm to 6-7 mm, and the pupas were still full (in the stage of pupal metamorphosis) (Figure 2).

**Figure 3:**
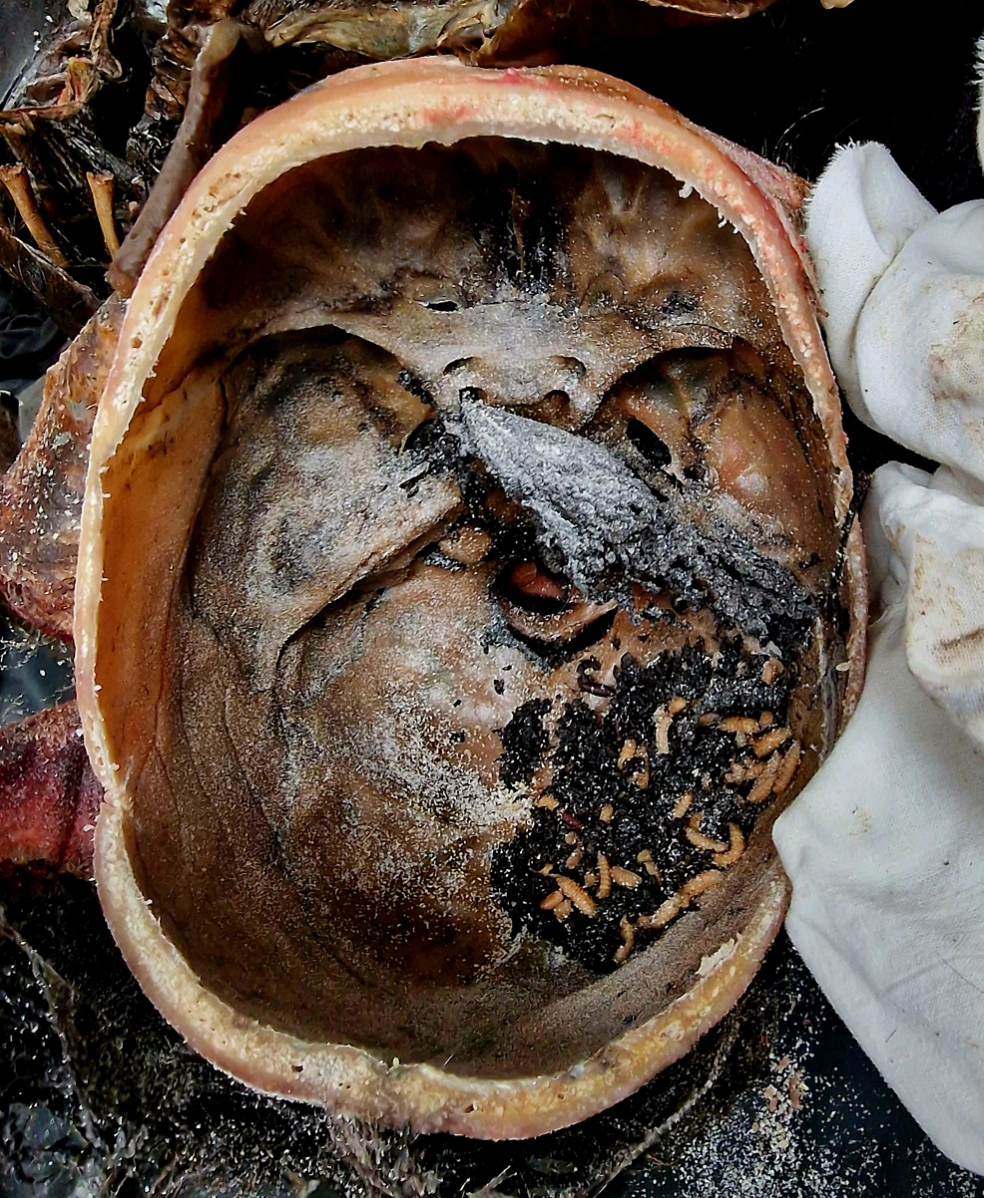
Internal examination of the skull – empty skull cavity with small deposition of maggots and pupas on the right side

**Figure 4:**
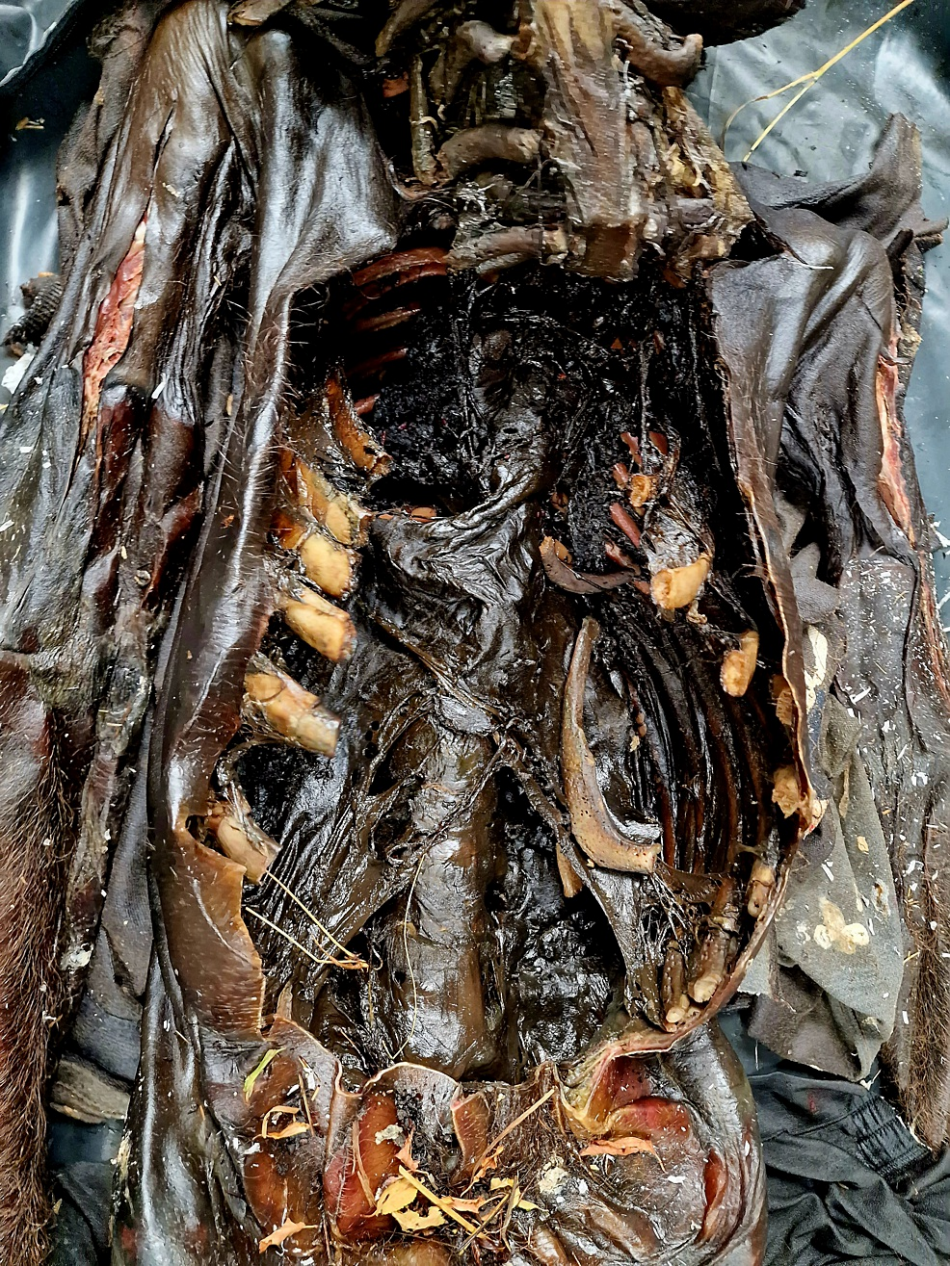
Internal examination of the thoracic and abdominal cavity – internal organs are in the form of dark brownish to blackish structureless masses and membranes

The internal examination of the body showed that the internal organs in the cranial, thoracic, and abdominal cavities had decayed into dried, brownish-black masses (Figures 3, 4).

**Figure 5:**
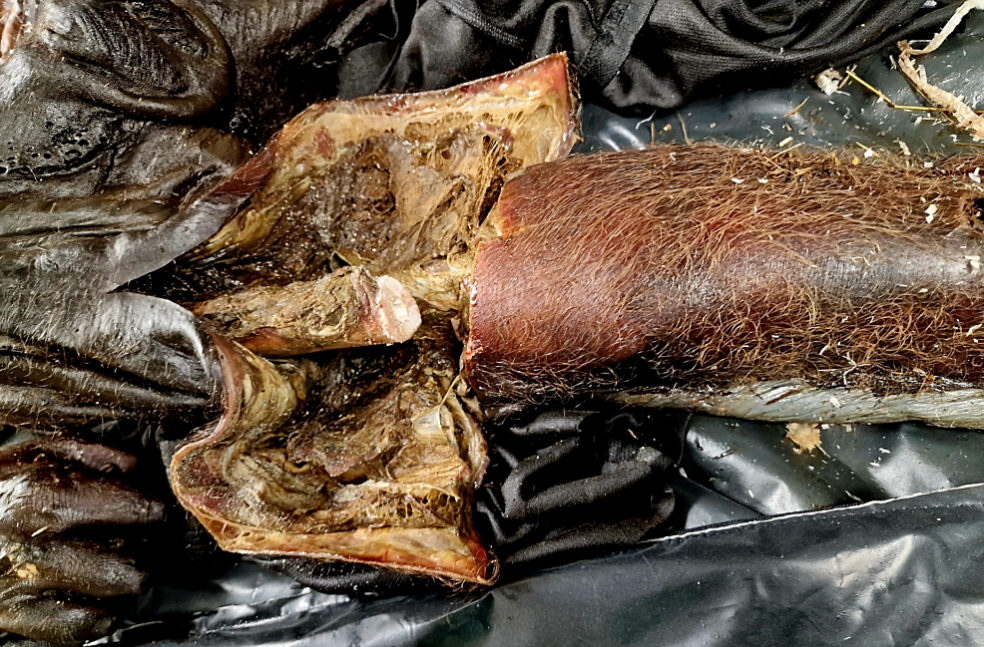
Additional cuttings of the soft tissues on the extremities – reduction of the fat tissue and desiccation of the muscles

Additional cutting was performed on the extremities, and no traumatic injuries were found. The subcutaneous fat tissue was reduced to almost entirely missing. The muscles were bright in color and dried (Figure 5).

Small pieces of the masses formed from the internal organs were taken for toxicology analysis, which was negative for the presence of drugs. A fragment from the right femur was taken for DNA analysis, which confirmed the deceased's identity. The cause of death was unknown. It was concluded that it is not associated with traumatic injuries. We cannot exclude the possibility of alcohol intoxication or complications related to its chronic abuse. Still, we cannot prove it without proper material for toxicological analyses and internal organs. A complete mummification of the corpse had occurred for a 16-day period.

Over the 16 days, the temperature ranged between 16°C and 33°C, with relative humidity for August at 52% and average solar radiation of 257.9 Wm^-2^. The wind in Sofia during August blows at an average speed of 8.1 mph (13.03 km/h).

## Discussion

Immediately after the death of a person, the decomposition process begins with the onset of autolysis. The last is associated with self-digestion - intracellular compounds are spilled into the surrounding tissues, facilitating the proliferation and distribution of the internal bacterial population [5]. The process of putrefaction goes through three phases - formation of gases and softening of the tissues, with further imbibition and decay. The putrefactive bloating gasses are formed due to the action of bacterial enzymes generated by different bacteria, especially Clostridium welchii, streptococci, Escherichia coli, and Proteus. These enzymes have destructing activity on tissue components - carbohydrates, fats, and proteins. Bacterial growth inside the corpse is moderated by several necessary conditions - warmth and moisture, leading to the formation of gases such as H2S, NH3, CO2, CH4, and others. Some of those gases connect with the hemoglobin in the blood and form the chemical compound called sulfhemoglobin, which is green in color, leading to the decomposed body's specific greenish discoloration. The putrefactive bacteria spread quickly in fluids, including the venous system. The last leads to staining the vessel wall with hemolyzed blood, giving the skin a marbled appearance [4,6,7]. The gases that have been formed lead to emitting a specific smell - products of the bacterial action, which attracts various insects or animals. Their activity, in combination with the bacterial, enzymatic, and physical action, leads to the breaking down of the complex organic body tissues into simpler inorganic compounds or elements, which macroscopically appear as softening, liquefaction and destructions of the soft tissues and internal organs [4,6,7].

Depending on the surrounding condition, the decomposition process might be fastened or cease and turn in an opposite direction to the preservation of the body [4,6]. Among the most common forms of conservative changes is mummification. Natural or artificial freezing, bog bodies, and saponification are other known forms. Extrinsic environmental factors, such as aridity, air circulation, solar radiation, and high temperatures, are the leading factors in mummification [8,9]. Such factors are known as mummification-inducing, and the following criteria must be met: a 24-hour maximum temperature > 30°C, an average solar radiation > 600 Wm^-2^, an average daytime humidity < 50%, and a windy day (daytime wind speed/gust measures of 20-30 mih^-1^, 32.1948.29 kmh^-1^) [4]. Days wherein not all of the mummification-inducing criteria are present but are close to those mentioned above are known as near-mummification-inducing. According to Finaughty and Morris, those are days where at least three of the four mummification-inducing criteria are present, or two of the thermal, humidity, and solar radiation criteria, plus at least two consecutive windy days immediately preceding the day in question [4]. Those specific ambient conditions will delay and block the bacterial action and the invasion of the insects, which will again reduce the speed of decomposition. Due to the evaporation of the bodies' liquid compounds, the mummified skin becomes leathery, parchment-like in appearance, with a bright yellow to dark brownish-to-black discoloration. The internal organs usually become dry, structureless masses and membranes [3,10]. The complete mummification of the human body is a time-consuming process and usually takes several weeks to 6-12 months [2,4]. In the case presented, it is seen that environmental factors are not needed for mummification. The measured values for August in Sofia's temperature, humidity, wind speed, and solar radiation are close to near-mummification-inducing. We assume that the movement of the trains since he was found next to a railway line, could provoke an additional “windy condition,” which could help in the fastening of the process.

One of the most challenging tasks for a forensic pathologist or medical examiner is estimating the postmortem interval or the time since death, which is even more problematic in cases of decomposed bodies and mummified ones. As mentioned earlier, different types of insects are attracted to dead bodies and invade them soon after. Most commonly in Bulgaria are the flies - Musca domestica, Calliphora vicina, Lucilia caesar, and Sarcophaga carnaria. They have a complete life cycle of egg, larva, pupa, and adult stages [11]. The life cycle of the flies could give a clue about the approximate time since death since each of its stages corresponds to a specific time interval, and the science that studies this process is forensic entomology [11]. As the process of decomposition or mummification depends on the environment and many other factors, the life cycle of the insects could be fastened or ceased by them [10,11]. In the studied case, it was obvious that the specific conditions were almost entirely stopping the insect invasion and development since there were just a few larvae and pupas when they were usually hundreds and thousands.

For our atmospheric and geographic conditions, the flies' whole life cycle varies from 14 to 18 days for the different fly species. The formation of the pupal stage starts approximately seven to 10 days following egg laying [12]. In the case presented, the body was covered with different-sized maggots and pupas in the stage of metamorphosis. These findings show that the whole life cycle of the flies has not been complete, which shows that the death occurred approximately less than 18 days before the body finding, which supports and corresponds to the given information that the deceased was last seen alive approximately 16 days earlier on the 18th of August.

There are just a few reported cases in the literature with rapid, also known as precocious mummification [2,4,13]. It is an extremely rare event, especially where the typical conditions for this process are not met.

## Conclusions

Precocious mummification is an infrequent phenomenon in nontropical or equatorial areas. Determining the postmortal interval is a challenging task, especially in cases of decomposed or mummified bodies. The presented case shows a complete natural mummification of the human body over just 16 days. The illustrated case will enrich the literature and will raise awareness of the possibility of fast-occurring mummification.

## References

[1] Ikram S (2010). Mummification. UCLA Encyclopedia of Egyptology.

[2] Marella GL, Perfetti E, Manciocchi S, Arcudi G (2013). A case of "precocious" mummification. J Forensic Leg Med.

[3] Radanov S, Stoev S, Davidov M, Nachev S, Stanchev N, Kirova E (1992). A unique case of naturally occurring mummification of human brain tissue. Int J Legal Med.

[4] Finaughty DA, Morris AG (2019). Precocious natural mummification in a temperate climate (Western Cape, South Africa). Forensic Sci Int.

[5] Mayer R (2011). Embalming: History, Theory, and Practice. https://www.google.co.in/books/edition/Embalming_History_Theory_and_Practice_Fi/SAV2ag_GcM4C?hl=en.

[6] Vass AA, Barshick SA, Sega G, Caton J, Skeen JT, Love JC, Synstelien JA (2002). Decomposition chemistry of human remains: a new methodology for determining the postmortem interval. J Forensic Sci.

[7] Campobasso CP, Di Vella G, Introna F (2001). Factors affecting decomposition and Diptera colonization. Forensic Sci Int.

[8] Ceciliason AS, Käll B, Sandler H (2023). Mummification in a forensic context: an observational study of taphonomic changes and the post-mortem interval in an indoor setting. Int J Legal Med.

[9] Pinheiro J (2006). Decay process of a cadaver. Forensic Anthropology and Medicine.

[10] Harding BE, Wolf BC (2015). The phenomenon of the urban mummy. J Forensic Sci.

[11] Sharma R, Garg RK, Gaur JR (2015). Various methods for the estimation of the post mortem interval from Calliphoridae: a review. Egypt J Forensic Sci.

[12] Radanov S, Doichinov I, Lisaev P, Stanchev N, Christov S (2006). Forensic medicine and medical deontology. (Article in Bulgarian). Siela.

[13] Kashimura S, Umetsu K, Ikeda N, Suzuki T, Oumi M, Hanaya S (1984). On a cadaver mummified within 25 days. Japan J Leg Med.

